# Development of a Tool to Identify Poverty in a Family Practice Setting: A Pilot Study

**DOI:** 10.1155/2011/812182

**Published:** 2011-05-26

**Authors:** Vanessa Brcic, Caroline Eberdt, Janusz Kaczorowski

**Affiliations:** Department of Family Practice, Faculty of Medicine, University of British Columbia, 3rd floor David Strangway Building, 5950 University Boulevard, Vancouver, BC, Canada V6T 1Z3

## Abstract

*Objective*. The goal of this pilot study was to develop and field-test questions for use as a poverty case-finding tool to assist primary care providers in identifying poverty in clinical practice. *Methods*. 156 questionnaires were completed by a convenience sample of urban and rural primary care patients presenting to four family practices in British Columbia, Canada. Univariate and multivariate logistic regression analyses compared questionnaire responses with low-income cut-off (LICO) levels calculated for each respondent. *Results*. 35% of respondents were below the “poverty line” (LICO). The question “Do you (ever) have difficulty making ends meet at the end of the month?” was identified as a good predictor of poverty (sensitivity 98%; specificity 60%; OR 32.3, 95% CI 5.4–191.5). Multivariate analysis identified a 3-item case-finding tool including 2 additional questions about food and housing security (sensitivity 64.3%; specificity 94.4%; OR 30.2, 95% CI 10.3–88.1). 85% of below-LICO respondents felt that poverty screening was important and 67% felt comfortable speaking to their family physician about poverty. *Conclusions*. Asking patients directly about poverty may help identify patients with increased needs in primary care.

## 1. Introduction

Tremendous advances have been made in health care delivery; however, poverty still has a profound impact upon the health of many patients [1–4]. Poverty is recognized as one of the most significant determinants of health, both as an independent risk factor and a predictor of morbidity for many chronic conditions [1, 2, 4–10]. Family physicians are well positioned to address these needs from within a patient-centered primary care model and on a population level [11, page 1651] [12–14]. 

Many family physicians recognize the ongoing impact of poverty upon their patients' lives; however, they often feel ill equipped to address these issues in a systematic way [15].

The first step is to identify those affected by poverty and its associated poor health outcomes. In this context, a clinician might consider “case finding for poverty”, for instance, in new patient visits, periodic health exams, or as they see fit. This would introduce this important determinant of health into the clinical encounter and facilitate better patient-centered care for those in need while helping physicians identify disparities within their practice populations.

The concept of poverty case finding faces several challenges, such as an increased clinical workload, inappropriate financial compensation for additional care required, and investment in community services to support the needs of this higher-risk group. Despite these challenges, disparities left unaddressed will lead to further adverse patient outcomes and increased costs in the long term [16, 17].

Case finding for poverty in clinical practice creates an opportunity to address a patient's unique needs while working towards more equitable resource distribution within a practice population [18]. In Canada, health inequities have been studied primarily on the neighbourhood level, which often determines health service provision for clustered disadvantaged populations such as Vancouver's Downtown Eastside. However, for communities or practice populations with a diverse socioeconomic makeup, this leads to an ecological fallacy where population characteristics are attributed to an individual [19]. This may be harmful for poor patients attending a primary care clinic in mixed or higher income neighbourhoods. A poverty case-finding tool employed in the clinical encounter provides the foundation for targeted interventions to reduce effects of poverty and risks of adverse health outcomes in low-income patients (Box 1). 

## 2. Methods

A literature review was conducted (MEDLINE, EMBASE, CINAHL, Web of Science, PsycINFO, HAPI) to identify previously validated social determinant questionnaires. A questionnaire was developed including direct and surrogate markers of poverty; items were selected from previously validated studies or reviewed by a panel of physicians and doctoral research experts working in the field and targeted to a Grade 8 reading level [31–37]. Sufficient demographic data was collected to assess respondents' income status: estimated yearly household income, postal code, and number of people per household. Four questions assessed respondent levels of comfort and perceived importance of proposed case-finding questions. The study design and questionnaire were approved by the University of British Columbia Ethics Committee. 

Between February and April 2009, questionnaires were completed by primary care patients in waiting rooms of four university-affiliated clinics in one rural and one urban centre in British Columbia, Canada. Equal samples of rural and urban, poor and wealthy respondents were sought, following the principle of maximum variation in sampling. A convenience sample of 100 questionnaires was required in order to ensure a margin of error of less than 10%. Inclusion criteria were the ability to read and write English and age over 19 years. Students were excluded from participating. Participants were alerted to the study by posters in the waiting rooms and direct offers by front desk staff. Participants were provided with an information letter about the study explaining anticipated benefits and harms; consent was confirmed by the completion of the questionnaire. 

Using the results of the written questionnaire, proposed case-finding questions were correlated with demographic data. The LICO (low-income cut-off) and LIM (Low Income Measure) were calculated for each respondent based on demographic data collected. These are both measures used by Statistics Canada to identify individuals below the “poverty line." The LICO uses calculations of family and community size to estimate the “income threshold at which families are expected to spend 20 percentage points more than the average family on food, shelter, and clothing” [38, 39]. The LIM uses family composition to determine a poverty line “set at 50% of adjusted median family income” [38, 39]. The two measures were compared and correlated; based on this correlation, the LICO was chosen as the gold standard measure of poverty against which responses to the proposed poverty case-finding questions were compared. 

We divided the respondents into 2 groups: above and below LICO. The answers to each proposed case-finding question, if measured by a likert scale, were recoded as binary outcomes. The sensitivity and specificity of each question to predict LICO status were calculated in a series of two-by-two tables. A multivariate stepwise logistic regression method employed likelihood ratios to identify which combination of questions was best predictor of whether individuals were above or below LICO. A *P* value of less than .05 was considered statistically significant in all of our analyses. Patient views on poverty case finding were reported in two likert scale questions; responses were compared to optional, open-ended qualitative comments which were read by the investigators to provide further insight into responses.

## 3. Results

One hundred and fifty six questionnaires were collected: 75 in Golden, BC (population 4500) and 81 in Greater Vancouver, BC (population 2.1 million). Of these, 145 had sufficient data for calculation of the LICO and LIM and inclusion in the subsequent analysis. A Cohen's kappa of 0.925 reflects the strong correlation between these two measures of poverty, and the LICO was chosen for the remainder of the analysis.

Table 1 shows the demographic characteristics of the sample cross-tabulated with income status calculated as above or below LICO. Of particular interest is that 84% of respondents below the LICO were “single” and 45% did not own a telephone. Aboriginal ethnicity, educational attainment, and access to extended health insurance were similar in both the above-and below-LICO groups. Six percent of respondents (*N* = 10) selected “don't know” when asked to estimate their yearly household income; no respondents left the question blank. 

A univariate analysis was conducted; in identifying best questions for poverty case finding, below LICO status was considered a positive outcome, and a positive response to a case-finding question was considered a positive risk factor for the outcome. Sensitivity and specificity were calculated for each of the questions, and likert scales were collapsed to facilitate analysis (Table 2). All results calculated were statistically significant (*P* < .05). Three questions about job insecurity were excluded from the analysis as preliminary calculations indicated that these were poor predictors of LICO status. The best performing question was (Table 2: Q7) “Do you (ever) have difficulty making ends meet at the end of the month?” (sensitivity 98%; specificity 60%; OR 32.3, 95% CI 5.4–191.5).

A stepwise multivariate analysis of the proposed case-finding questions was conducted to determine if a combination of questions would perform better than any single question. Three questions were identified (Table 2: Q1, Q4, Q7), with a combined specificity of 94.4% and a sensitivity of 64.3%. Their combined odds ratio was 30.2 (95% CI 10.3–88.1).

When asked “How difficult has it been for you to get health care when you needed it in the last year?” none of the respondents above LICO found it “very difficult,” compared to 37% of respondents below LICO who found it very or somewhat difficult obtaining healthcare. When asked “… what kind of help did you need that you did not receive?” below-LICO respondents identified the following from a list of issues taken from the Canadian Community Health Survey [39]: information about service availability, mental illness and its treatments; therapy or counselling; help with personal relationships, alcohol, drugs, and addictions. For these areas, the below-LICO respondents were 4 to 7 times more likely to answer that they had difficulty getting help. 

The majority of below-LICO respondents (85%, *N* = 40) felt that poverty case-finding was very or somewhat important, and 67% (*N* = 33) felt very or somewhat comfortable speaking to their family physician about poverty-related issues. Sixty-five respondents volunteered comments when asked if any case-finding questions were “inappropriate” or “especially important” to be asked in a primary care setting. Four respondents identified that asking about a patient's source and amount of income was inappropriate. Thirty-seven respondents stated that none of the questions were inappropriate. Four respondents replied that the questions were acceptable if asked “in an appropriate way.” When asked to identify “especially important questions,” 12 respondents replied either that all questions were important or identified three or more topic areas as especially important, including access to food, housing, and health care; finances; mental health and coping; ability to pay for medications. 

## 4. Discussion

In the development of this study, it was debated whether to test known indicators of income poverty (social determinants of health such as food, job, and housing security) or other indicators directly relevant to family practice (e.g., access to a telephone or extended health insurance). As shown in Table 1, these latter indicators performed poorly, justifying the choice of the former in developing a poverty case-finding tool. We suspect that the high prevalence of extended insurance coverage among low-income respondents can be accounted for by government-funded insurance programs available to respondents receiving disability or income assistance. In general, rural respondents reported more additional health insurance, which may be due to coverage offered by major employers in Golden, BC, including forestry. Interestingly, education had no predictive value of respondents' above or below LICO status. This correlates with research suggesting that education is a less sensitive indicator of poverty as it is a fixed variable with larger gradations and variability in measurement compared to income [40]. 

A potential sample bias exists as all respondents were surveyed in clinic waiting rooms, demonstrating an ability and willingness to access care. Despite this, unmet needs were reported in the below-LICO group. Respondents' comments further suggested that access may still be a concern for some low-income patients already accessing primary care services. These areas of need are well correlated with previous research describing similar conditions that both influence and are a consequence of poverty [41]. These unmet needs provide fertile ground for intervention by a primary care practitioner.

### 4.1. Univariate Analysis

In the assessment of case-finding questions, the authors favoured sensitivity over specificity in the univariate analysis, as it is more useful for family physicians to accurately identify poverty as opposed to ruling out “wealth.” We also excluded questions which had a highly subjective response as these were not felt to be reliable. 

There is robust evidence identifying food security as a reliable surrogate marker of poverty [42]. This study correlates well with this literature in that the lack of money to pay for food and hunger related to lack of food were good predictors of below-LICO status (Table 2: Q1, Q3). Comments volunteered by respondents further illustrated the link between food insecurity and poor health. Sensitivity and specificity were comparable for Q1 and Q3 which used slightly different wording to assess food security. While Q1 had a marginally higher sensitivity, the simplified wording of Q3 is much more applicable for use in a family practice.

An important element of this study is the comparison of direct and indirect markers of poverty: Can we ask patients directly about poverty? Palliative care research suggests that patients often prefer that physicians speak to them directly about difficult issues [43, 44]. Our study supports this concept as it applies to poverty, as the best-performing question in the univariate analysis was “Do you (ever) have difficulty making ends meet at the end of the month?” (Table 1: Q7). Overall, respondents felt that asking about poverty-related issues in primary care is important. One participant offered that these questions “can be very important, especially when getting help with special diets or medications.” Another stated “I think it is very important that the doctor be aware of their patients' financial situation especially when it comes to prescriptions and their cost.”

Asking patients “How difficult is it to make ends meet?” (Q8) had poorer sensitivity compared to Q7 “Do you have difficulty...” (78% versus 98%: Table 2). This may be attributed to the relatively subjective nature of the former question. The high sensitivity of Q7 (98%; OR 32.3; 95% CI 5.4–191.5) could also be explained by the collapse of likert scale responses. Assigning a positive response to those who had always, most of the time, sometimes, or rarely had difficulty making ends meet included all respondents who had *ever* had difficulty and may reflect the dynamic or fluctuant nature of income poverty [40]. In developing direct poverty case-finding questions, it was impossible to avoid the use of colloquial terminology to describe poverty. Despite the high performance of Q7, “making ends meet” may be difficult to understand by patients for whom English is a second language. “Paying your bills” may be a more accessible phrase to be tested clinically or in future research.

### 4.2. Multivariate Analysis

The multivariate analysis identified three questions to form a best-performing multi-item poverty case-finding tool (Table 2: Q7, Q1, Q4). Q4 was included despite a low sensitivity of 39% in the univariate analysis. Its value in a multi-item case-finding tool can be explained by its high specificity (96% of respondents above LICO had never slept outside, in a shelter, or in a place not meant for sleeping) and low correlation with Q7 and Q1. The sensitivity and specificity of the multi-item tool were 64.3% and 94.4%, respectively. These results are superseded, however, by the high sensitivity (despite poorer specificity) of Q7 alone as well as comparable odds ratios between Q7 and the three-item tool (OR 32.3 versus OR 30.2). The authors, therefore, suggest the use of the single direct poverty case-finding question identified with the possibility of adding supplementary questions at the clinician's discretion.

### 4.3. Limitations

This study has several limitations. First and foremost, we adopted a binary definition of poverty for the purpose of this study; however, we recognize that poverty is a dynamic variable which presents along a continuum. In addition, both the LICO and LIM are generally considered to be poor estimates of the “poverty line.” There are likely some individuals who are classified as below LICO who have a good quality of life and do not “suffer” from poverty; there are likely more individuals who are classified as above LICO who have significant difficulties making ends meet. Self-reported income is also confounded by recall bias, social desirability bias, lack of control over and thus knowledge of income, and fear of disclosure of income. Also, the most recent LICO and LIM data were from 2007 and 2006, respectively, which may imperfectly correspond to reported incomes in 2009.

It is significant to note that we excluded individuals who could not read and write English for the pilot testing of case-finding questions. Given the growing body of literature demonstrating the association between low literacy, deprivation, and poor health outcomes [45], this exclusion likely caused an important segment of the population to be missed in this study. However, future research anticipates testing this question orally in a clinical setting in order to study its use in patients with all literacy levels. Furthermore, this study was conducted in Canada where universal health coverage is available to all citizens. The impact of poverty case finding and applicability of these results may vary significantly in different health care systems. Finally, a convenience sample was selected for this pilot study for the purpose of validating the questions. As this was not a representative sample, the results are not yet generalizable without further study.

## 5. Conclusion

The purpose of this study was to create an evidence-based tool for family physicians to identify poverty in primary care. Asking patients directly about poverty may help identify patients with increased needs in a practice population. The question “Do you (ever) have difficulty making ends meet at the end of the month?” not only was acceptable to patients but also had the highest sensitivity (98%) and Odds Ratio (32.3) of all questions tested in this study. A well-performing multi-item tool was also identified with good sensitivity and specificity, indicating that surrogate markers of poverty could be effectively used as adjunctive case-finding measures; these findings correlate with previous research demonstrating that food insecurity and recent homelessness are robust predictors or indicators of poverty. 

This pilot study aims to facilitate the recognition of health disparities in a family practice population by providing a foundation for further research. It also suggests that openly discussing poverty in the clinical encounter is likely important and acceptable to most patients. Next steps will involve testing this question orally in a representative sample and comparing results to other markers of disparity including, for example, health literacy status. Given the enormity of the epidemic of poverty, the development and application of such a tool is long overdue.

## Figures and Tables

**Box 1:**
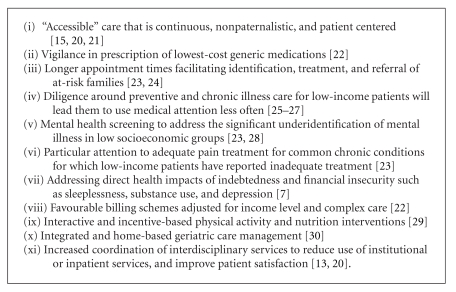
Poverty interventions in family practice.

**Table 1:** Demographics.

	Above LICO no. (%)	Below LICO no. (%)	Total valid no.
Overall	94 (65.0%)	51 (35.0%)	145
Above LIM	90 (98.9%)	1 (1.1%)	91 (62.7%)
Below LIM	4 (7.4%)	50 (92.6%)	54 (37.2%)
Male	35 (37.6%)	27 (54.0%)	68 (43.6%)
Female	58 (62.4%)	22 (44.0%)	84 (53.8%)
Rural	52 (76.5%)	16 (23.5%)	68 (47.5%)
Urban	40 (53.3%)	35 (46.7%)	75 (52.4%)
Married/ common law	62 (66.7%)	8 (15.7%)	73 (47.4%)
Sep/divorced/ widow/single	31 (33.3%)	43 (84.3%)	24 (15.6%)
Aboriginal	9 (9.8%)	5 (10.4%)	14 (9.3%)
High school	38 (41.3%)	23 (46.9%)	66 (43.7%)
College/ university	49 (53.3%)	25 (51.0%)	76 (50.3)
Has extra insurance	72 (77.4%)	33 (64.7%)	105 (72.9%)
Owns phone	90 (96.8%)	27 (52.9%)	117 (81.3%)
No phone	3 (3.2%)	23 (45.1%)	26 (18.1%)

**Table 2:** Results of univariate analysis for proposed case-finding questions.

Survey questions*	Above LICO no. (%)	Below LICO no. (%)	Total valid no. (%)	Sensitivity % (95% CI)	Specificity % (95% CI)
(Q1) In the past year, was there any day when you or anyone in your family went hungry because you did not have enough money for food? *Answer: Yes *	5 (5.6%)	32 (64%)	37 (25.8%)	64 (55.2–69.4)	94.6 (89.9–97.5)
(Q2) Can you afford to eat balanced meals? *Answer: Rarely/Never *	1 (1.1%)	13 (25.5%)	14 (9.6%)	25.5 (19.2–27.1)	98.9 (95.5–99.8)
(Q3) After paying your monthly bills, do you typically have enough money left over for food? *Answer: No *	9 (9.9%)	27 (60%)	36 (26.4%)	60.6 (49.6–68)	90.1 (85–94.1)
(Q4) In the last month, have you slept outside, in a shelter, or in a place not meant for sleeping? *Answer: Always *→* Rarely *	4 (4.3%)	20 (39.2%)	24 (16.5%)	39.2 (31.1–43.8)	95.7 (91.4–98.2)
(Q5) Do you ever worry about losing your place to live? *Answer: Always *→* Rarely *	36 (38.3%)	44 (86.3%)	80 (55.1%)	86.3 (76.4–92.8)	61.7 (56.4–65.3)
(Q6) How many times have you moved in the last year? *Answer: 3 or more times *	3 (3.2%)	17 (33.3%)	20 (13.7%)	33.3 (25.8–37)	96.8 (92.7–98.9)
(Q7) Do you have difficulty making ends meet at the end of the month? *Answer: Always *→* Rarely *	55 (59.8%)	48 (98%)	103 (73.0%)	98 (90.4–99.6)	40.2 (36.2–41.1)
(Q8) Considering your current income, how difficult is it to make ends meet? *Answer: Difficult *	25 (27.2%)	38 (77.6%)	63 (44.6%)	77.6 (66.9–85.9)	72.8 (67.2–77.3)
(Q9) Do you have enough money to get by? *Answer: Rarely/Never *	5 (5.4%)	18 (36.7%)	23 (16.3%)	36.7 (28.2–42.2)	94.6 (90–97.5)

*Three job security questions present in survey were poorly performing and excluded from full analysis.
